# Glutamine metabolic reprogramming in hepatocellular carcinoma

**DOI:** 10.3389/fmolb.2023.1242059

**Published:** 2023-08-11

**Authors:** Yanyan Ye, Bodong Yu, Hua Wang, Fengming Yi

**Affiliations:** ^1^ Department of Ultrasound, The Second Affiliated Hospital of Nanchang University, Nanchang, China; ^2^ The Second Clinical Medical College of Nanchang University, The Second Affiliated Hospital of Nanchang University, Nanchang, China; ^3^ Jiangxi Medical College, Nanchang University, Nanchang, China; ^4^ Department of Oncology, The Second Affiliated Hospital of Nanchang University, Nanchang, China; ^5^ Jiangxi Key Laboratory of Clinical and Translational Cancer Research, Nanchang, China

**Keywords:** hepatocellular carcinoma, glutamine metabolic reprogramming, metabolic targeting therapy, mTORC, glutamine-related metabolites

## Abstract

Hepatocellular carcinoma (HCC) is a lethal disease with limited management strategies and poor prognosis. Metabolism alternations have been frequently unveiled in HCC, including glutamine metabolic reprogramming. The components of glutamine metabolism, such as glutamine synthetase, glutamate dehydrogenase, glutaminase, metabolites, and metabolite transporters, are validated to be potential biomarkers of HCC. Increased glutamine consumption is confirmed in HCC, which fuels proliferation by elevated glutamate dehydrogenase or upstream signals. Glutamine metabolism also serves as a nitrogen source for amino acid or nucleotide anabolism. In addition, more glutamine converts to glutathione as an antioxidant in HCC to protect HCC cells from oxidative stress. Moreover, glutamine metabolic reprogramming activates the mTORC signaling pathway to support tumor cell proliferation. Glutamine metabolism targeting therapy includes glutamine deprivation, related enzyme inhibitors, and transporters inhibitors. Together, glutamine metabolic reprogramming plays a pivotal role in HCC identification, proliferation, and progression.

## 1 Introduction

Hepatocellular carcinoma is a heterogeneous and lethal disease with increasing incidence and mortality globally (Wang et al., 2021; Llovet et al., 2022a). More than 80% of HCC occurs in Eastern Asia and sub-Saharan Africa with limited medical resources (Yang et al., 2019). Genetic predisposition, risk factors, tumor microenvironment (TME), and underlying disease promote the malignant hepatocyte transformation, development, and progression (Yang et al., 2019; Llovet et al., 2021). The management of HCC is according to the tumor stages with mostly applicated Barcelona Clinic Liver Cancer (BCLC) staging system. Briefly, curative therapeutics, including liver resection, transplantation, and tumor ablation, are selected for early-stage patients; Transarterial chemoembolization (TACE) is suitable for intermediate stages; systemic therapies are candidates for advanced settings, whereas best supportive care is most appropriate for end-stage of HCC (Llovet et al., 2021). Extended morphometric and biological criteria applied for surgery and liver transplantation for HCC are confirmed to promote the overall survival of some selected patients. However, 22%–25% of patients are recurrent after resection in 10 years, and 50%–70% are recurrent after transplantation (Vibert et al., 2020). For patients in advanced stages, systemic treatment is recommended as the standard of care. Molecular targeted monotherapy, including sorafenib or lenvatinib in the first line, and regorafenib, cabozantinib, or ramucirumab in the second line, has been confirmed to improve clinical outcomes with limited median overall survival (Llovet et al., 2018; Faivre et al., 2020). Immune-checkpoint inhibitor (ICI)-anti-programmed cell-death protein (ligand)-1 (PD-[L]1), is proven to be effective in the treatment of HCC. However, the ORR is limited to 10%–20% of HCC patients for monotherapy. The combination of the anti-angiogenic drug bevacizumab and immune-checkpoint inhibitor atezolizumab has already reshaped to be the standard first-line treatment regimen (Llovet et al., 2022a), and the expected survival of HCC with advanced stage could reach up to more than 2 years (Finn et al., 2020; Reig et al., 2022). Other dual therapies combing ICIs with multi-kinase inhibitors are proven to be promising in clinical trials. Oncolytic virus immunotherapy, adoptive T-cell transfer, and anti-immunosuppressive environment strategies are under exploration with promising futures (Foerster et al., 2022).

Molecular classifications with molecular signatures, pathological features, genetic features, typical signaling pathways, epigenetic features, and immunological features will be helpful in precise treatment (Rebouissou and Nault, 2020; Llovet et al., 2022b). Mutation of Wnt/β-catenin is revealed in 35% of HCC patients; mounting strategies targeting the Wnt/β-catenin cascade have provided evidence in preclinical trials in recent decades (Xu et al., 2022). Tumor-associated exosomes are proven to shape the local and distant microenvironments of HCC initiation and development. The preclinical application of biomarkers, drug resistance, and treatment are under exploration (Wang et al., 2022). Preclinical studies depict that selective inhibiting tumor-promoting neutrophils, related signaling pathways, and chemotaxis are effective (Geh et al., 2022). Non-cellular components, including hypoxia, cytokines secreted by tumor stroma, and extracellular matrix, also play a pivotal role in forming the cancer stem cell niche in HCC, which might be potential clinical applications in the future (Lam and Ma, 2022).

Metabolic reprogramming has been frequently unveiled in HCC, such as tumor favors Warburg effect rather than oxidative phosphorylation, unbalanced lipid intake, and fatty acid mobilization causing high levels of circulating glucose and fatty acids, which induces alternative source of energy of cancer cells (Satriano et al., 2019). The elucidation of metabolic characteristics is promising in understanding or treating HCC.

## 2 HCC and metabolism

The liver plays a pivotal role in metabolic homeostasis. The oxygen gradient from periportal hepatocytes towards pericentral hepatocytes corresponds to a different function in the hepatic zonation. Periportal hepatocytes (zone 1) have a substantial oxygen supply from arterioles responsible for gluconeogenesis, albumin synthesis, amino acid (AA) catabolism, cholesterol synthesis, and β-oxidation, which need more ATP for energy supply. Pericentral hepatocytes (zone 3) serve glycolysis, glutamine synthesis, lipogenesis, and detoxification. The hepatocytes located between periportal and pericentral hepatocytes (zone 2) serve for iron metabolism and insulin-like growth factor (IGF) homeostasis (Li et al., 2021).

Otto Warburg first demonstrated that HCC tissue consumed glucose and converted it into lactate rather than untaken by mitochondria for the TCA cycle, even in the existence of sufficient oxygen, also termed aerobic glycolysis or the Warburg effect. Aerobic glycolysis in HCC results in more glucose uptake, faster ATP generation, and lactate production (Liberti and Locasale, 2016; Satriano et al., 2019). In addition, the Warburg effect also supports anabolic metabolism by providing the pentose phosphate, hexosamine, and glycerol pathways without preventing mitochondrial respiration. The decreasing of oxidative phosphorylation (OXPHOS) renders the reduction of reactive oxygen species (ROS). Aerobic glycolysis mediates proliferation, growth, immune evasion, invasion, migration, angiogenesis, and drug resistance in HCC (Alannan et al., 2020; Feng et al., 2020).

A higher rate of lipogenesis is a hallmark of cancer cells. HCC has demonstrated that the enhancement of the Warburg effect attributes to an increase in the level of β-oxidation by metabolomics studies. Lipid catabolism also provides energy to promote cancer cell proliferation and produces metabolites for biosynthesis to meet fast-growing tumors. Lipid metabolism reprogramming promotes abnormal gene expression and rewires many oncogeneses and metastasis-related pathways. Targeting lipid metabolism has the potential anti-tumor activity in preclinical studies (Alannan et al., 2020). Dysfunction of lipid metabolism, like nonalcoholic fatty liver disease (NAFLD), is one of the main risk factors for HCC. Treatment of NAFLD might have anti-tumor potential (Orabi et al., 2021). HCV protein has been validated to hijack the patients’ lipid and glucose metabolism by stimulating *de novo* lipogenesis, promoting synthesis of phospholipids and sphingomyelins, inhibiting mitochondria fatty acid oxidation, and hijacking the very low-density lipoprotein (VLDL) secretion pathway. HCV promotes hepatocellular carcinogenesis via crosstalk with metabolic dysfunction; it will boost oxidative stress, DNA damage, lipo-toxicity, cell death, and senescence in patients with adipose tissue dysfunction and insulin resistance (Leslie et al., 2022). Metabolic impairment might be the potential reason for HCV-related HCC early recurrence even after direct-acting antivirals (Reig et al., 2016). Cholesterol metabolisms play a double-edged sword in hepatocellular carcinoma. Cholesterol can not only induce ectopic fatty acids accumulation, reshape an immunosuppressive microenvironment, activate hepatic stellate cells, and influence membrane fluidity or protein function, to further promote tumorigenesis in HCC but also activate NK cell proliferation or recruitment, and promote CD44 translocation into lipid rafts, so that prohibit HCC (Zhou and Sun, 2021).

Other metabolism alternations such as proline metabolism, cysteine metabolism, nucleotide metabolism, urea cycle, hexosamine biosynthetic pathway, pentose-phosphate pathway, et al. are also validated in HCC(17, 26–28). Proline metabolism has been confirmed to enhance the tumorigenesis in liver cancer as two enzymes corresponding to proline biosynthesis are upregulated (pyrroline-5-carboxylate reductase (PYCR1), aldehyde dehydrogenase 18 family member A1 (ALDH18A1)), and one proline catabolic enzyme is downregulated (proline dehydrogenase (PRODH)) (Ding et al., 2021). Cysteine metabolism plays a pivotal role in sorafenib responses during HCC therapy (Byun et al., 2022), maintaining glutathione synthesis to protect HCC cells from ferroptosis (Hu et al., 2022).

Moreover, the interplays between metabolism and tumor microenvironment play crucial roles in the cancerous liver. It could be subclassified into antitumor immunometabolism and protumor immunometabolism. For instance, increased fatty acid synthesis and glycolysis in Th17 cells could enhance the production of IFN-γ, which will function as an antitumor effect; elevation of β-oxidation in tumor-associated macrophages promotes M2 macrophage polarization, which exerts as protumor function (Li et al., 2021). Hypoxia in the HCC also induces the activation of lactate metabolism, serine synthesis pathway and folate cycle, and adenosinergic metabolism to support the growth of tumors (Bao and Wong, 2021).

Glutamine is an indispensable energy fuel and nitrogen source for tumor initiation, survival, and progression; It functions not only as an energy resource but also biosynthesis, signaling pathway regulator, regulating ROS, and maintaining tumor microenvironment; and increased glutamine consumption is conserved in different cancers (Yang et al., 2021; Gyamfi et al., 2022; Ma et al., 2022). Herein, we will conclude the role of glutamine metabolic reprogramming in HCC.

## 3 Glutamine and cancer

Glutamine is a nonessential amino acid (NEAA) that can be synthesized *de novo* by glutamine synthetase. In contrast, the increased demand in tumors results in glutamine, a conditionally essential amino acid. Glutamine also functions as an intracellular exchange factor or deamidated to glutamate, an elemental carbon and nitrogen source, especially for glutamine-addicted cancer cells. Moreover, glutamine metabolism involves substantial biosynthesis, including anti-ROS glutathione/NADPH and lipids synthesis. In addition, glutamine is a nitrogen donor for hexosamine, asparagine, and nucleotide biosynthesis through aminotransferases (Altman et al., 2016; Yang et al., 2021).

**FIGURE 1:**
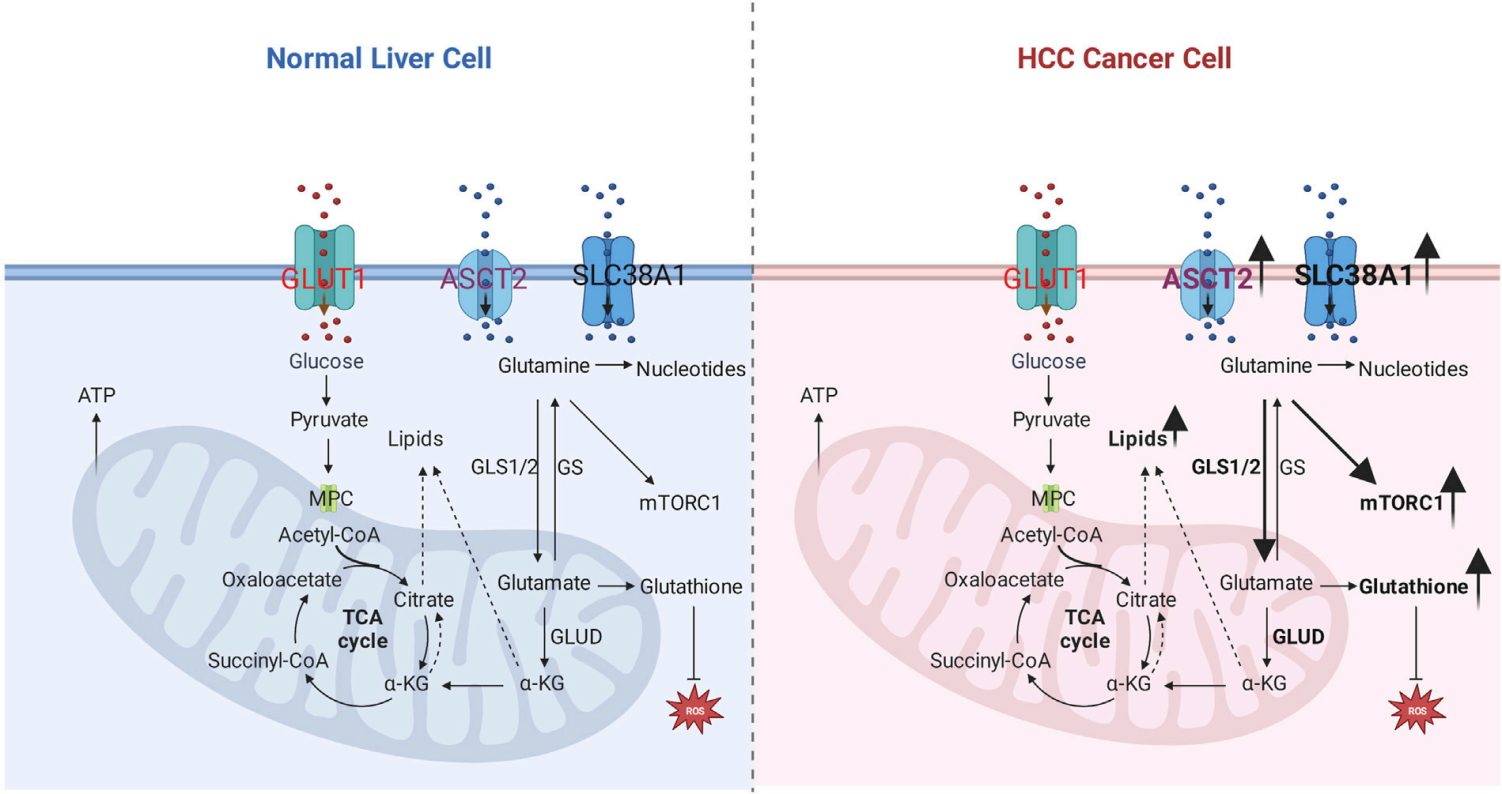
Glutamine metabolic reprogramming in hepatocellular carcinoma. Left: glutamine metabolism in the normal liver cell. Right: glutamine metabolism in hepatocellular carcinoma; the number of glutamine transporters increases, which results in more glutamine uptake by the cancer cell, related enzymes such as GLS1/2, GLUD are elevated to enhance sources for carbon and nitrogen, lipids, and nucleotides; improved glutamine uptake activates mTOR1 pathway and antioxidative effect.

Glutamine is a major substrate of the TCA cycle’s component; it participates in the biosynthesis of biomolecules, maintaining redox homeostasis, ATP generation, oxidative metabolism, and signaling pathway as one of the major nutrients. Moreover, it also provides the energy for activated or proliferative cells such as cancer cells and activated lymphocytes (Gyamfi et al., 2022). Glutamine metabolism also participates in essential biological processes, including nucleotide/amino acids/extracellular matrix synthesis, protein glycosylation/epigenetic modification, cellular redox balance, and autophagy (Fan et al., 2020). The primary process of glutamine metabolism includes the following steps: cells uptake glutamine by specific transporters (SLC1A5/ASCT2) and then convert glutamine to glutamate in mitochondria by glutaminase (GLS); subsequently, glutamate will convert to α-ketoglutarate (α-KG) as the main component of Tricarboxylic Acid cycle (TCA) by mitochondrial glutamate dehydrogenases (GLUD), α-KG mainly incorporates into TCA cycles to assist the production of NADPH and nucleotide synthesis. Furthermore, α-KG will be exported to the cytoplasm as a source of acetyl-CoA, which is the main substrate of fatty acid synthesis; Or convert to glutathione (GSH) to further stabilize the redox homeostasis (DeBerardinis et al., 2007; Zhang et al., 2017), and it also can transfer the amine group to another nonessential amino acid by transaminases. Besides, it can be subtracted of proline and glutathione biosynthesis (Yang et al., 2021); the details are shown in Figure 1.

Glutamine metabolism also plays a crucial role in the interplay between TME and tumor cells. The competition of glutamine consumption by immune cells and tumor cells results in the immune response from tumor-infiltrating T cells as glutamine deficiency. Moreover, the shortage of glutamine for tumor cells will induce the proliferation and activation of Treg cells, which function as an immunosuppressive effect (Cluntun et al., 2017; Fu et al., 2019; Edwards et al., 2021). However, cancer-associated fibroblasts (CAFs) rescue the glutamine-deficiency microenvironment by complementary secreting glutamine (Yang et al., 2016). Glutamine reprogramming also impacts other immune cells’ polarization or function (Ma et al., 2022).

Inhibition of glutamine metabolism has been confirmed to be promising in glutamine-addicted cancer cells, including glutamine analogs like DON, acivicin, and azaserine, glutamine transporter inhibitor GPNA and V-9302, GLS1/2 inhibitors (Shen et al., 2021). GLS1 plays a crucial role in cancer progression by converting glutamine to glutamate in mitochondria. It enhances tumor development, invasion, and migration by maintaining redox homeostasis, cellular energetics, and proliferative signaling pathway. GLS1 demonstrates higher expression in solid tumors such as stomach adenocarcinoma, head and neck squamous cell carcinoma, thymoma, testicular germ cell tumors, hepatocellular carcinoma, and colon adenocarcinoma, according to the TCGA database analysis. It is regulated by Myc, Retinoblastoma, and nuclear transcription factor-κB in cancer cells. GLS1 inhibitors, including DON, BPTES, 968, CB-839, UPGL00004, and ebselen, show promising anti-tumor effects for glutamine-dependent cancers (Yu et al., 2021). bis-2-(5-phenylacetamido-1,3,4-thiadiazol-2-yl) ethyl sulfide (BPTES) and CB-839, GLS inhibitors are confirmed to have anti-tumor effective; especially CD839 has been proved to possess the ability of antiproliferative activity in both solid tumors like pancreatic cancer and breast cancer (Gross et al., 2014; Biancur et al., 2017). However, drug resistance has been validated in targeting glutaminolysis. Numerous studies are trying to explore the treatments to conquer the drug-resistance, including the combination of GLS1 and GLS2 inhibitors; GLS1 inhibitors synergize with glutamate release blockage, targeting glutaminolysis accompanied with other clinical drugs like chemotherapy/molecular targeted therapy/immune therapy, and GLS inhibitors combined with other metabolic inhibitors (Shen et al., 2021; Lemberg et al., 2022). JHU-083 is a pro-drug proven to inhibit tumor growth and reshape the tumor immune microenvironment, promoting CD8+T activation and proliferation and decreasing immunosuppressive myeloid cells (Oh et al., 2020). Moreover, inhibition of glutaminolysis will induce the expression of PD-L1 in tumor cells, which indicates that the combination of anti-glutaminolysis and immune checkpoint blockade would have a synergistic antitumor effect (Byun et al., 2020). Glutamine uptake inhibitors(V-9302), glutamine antimetabolites(L-DON/JHU-083), and glutaminase inhibitors (CB-839) are confirmed to be effective in reshaping glutamine metabolism in immune cells and function as anti-tumor immune microenvironment (Ma et al., 2022). Glutamine transporter is upregulated in various tumors, such as SLC7A5, which is regulated by oncogene c-Myc. C-Myc or KRAS mutation also upregulated the expression of GLS(33, 50). MYC, SLC1A5, mTORC1, and glutaminase could be further utilized as a biomarker to recognize glutamine-addicted cancers (Yuneva et al., 2007; Wise and Thompson, 2010; Bhutia and Ganapathy, 2016). SLC1A5 is widely upregulated in tumors among 14 glutamine transporters (Bhutia and Ganapathy, 2016). V-9302, a glutamine transporter inhibitor targeting SLC1A5/ASCT2, has validated the effect of attenuating tumor cell proliferation and increasing the infiltration of CD8+T cells (Schulte et al., 2018; Pallett et al., 2021).

## 4 The components of glutamine metabolism as biomarkers of HCC

Forty-one glutamine metabolism (GM) associated genes are termed as GMScore from The Cancer Genome Atlas (TCGA) and the International Cancer Genome Consortium (ICGC) database. High GMScore indicates tumor growth and poor overall survival. In addition, High GMscore predicts a low response to immune checkpoint inhibitors (Ying et al., 2021). A study tries to depict the different gene expressions between poorly differentiated HCC cell lines and well-differentiated HCC cell lines from public databases. Metabolic-related gene analysis demonstrates that poorly differentiated cell lines profoundly rely on glutamine to fuel the TCA cycle (GLS, SLC1A5, SDHA). However, well-differentiated cell lines depend on glycolysis and glutaminolysis (Nwosu et al., 2018). The components of glutamine metabolism, including metabolic enzymes, metabolites, and metabolite transporters, demonstrate high sensitivity and specificity in diagnosis, relapse monitoring, and stage prediction.

### 4.1 Glutamine synthetase

Glutamine synthetase (GS) is the feature of Wnt/β-catenin pathway activation, expressed in the pericentral hepatocytes, and elevated GS indicates cell proliferation in tissues (Cadoret et al., 2002; Austinat et al., 2008; Bioulac-Sage et al., 2009; Sohn et al., 2018; Ruiz de Galarreta et al., 2019; Tao et al., 2021; Hamaguchi et al., 2022). Liver tumors with different β-catenin activation levels demonstrate distinct tumor phenotypes. Highly activating β-catenin with CTNNB1 mutation types is associated with malignant transformation and intense pattern of GS staining; However, weak mutations display more frequently for hepatocellular adenoma (HCA) (Rebouissou et al., 2016). Activated β-catenin in HCC patients predicts better survival and less sorafenib resistance than inactive ones. The potential mechanism of the β-catenin effect might be mediated by autophagy via increasing GS (Sohn et al., 2018).

Glutamine synthetase could distinguish atypical nodules, early diagnosis, and invasion of HCC. For instance, GS is considered to have high specificity and sensitivity to the differential diagnosis of HCC and dysplastic nodules (Coral et al., 2021). Glypican-3, heat shock protein 70, and GS are utilized to distinguish a <2 cm hepatocellular lesion without classic radiological characters of HCC with cirrhosis by immunochemistry (IHC) or designed RNA probes (Tremosini et al., 2012; Di Tommaso and Roncalli, 2017; Bakheet et al., 2020). GS and glypican3 staining are sensitive and specific to HCC compared to metastatic cancer, benign hepatocellular lesions, and cirrhosis. They are associated with large tumor sizes and poorly differentiated specimens (Wasfy and Shams Eldeen, 2015). GS positive staining has 43.9%–100% sensitivity for HCC compared with cirrhotic nodules (Dal Bello et al., 2010; Long et al., 2011; Shin et al., 2011; Witjes et al., 2013; Uthamalingam et al., 2018). The sensitivity and specificity of IHC GS staining for the early stage of HCC are 50% and 90%, respectively (Dal Bello et al., 2010); the sensitivity and specificity of GS in distinguishing low-grade HCC from hepatocellular adenoma (HCA) are 80% and 50%, respectively (Lagana et al., 2013). GS has upregulated in steatohepatitis hepatocellular carcinoma, which is validated by RNAseq or immunochemistry (Van Treeck et al., 2021). However, another study shows that GS is expressed in relatively few tumors induced by DEN or metabolic dysfunction associated with fatty liver disease (MAFLD) in mice (Kurosaki et al., 2021). HCC with steatohepatitis has a low incidence of glutamine synthetase overexpression and nuclear accumulation of β-catenin (Ando et al., 2015). GS is highly expressed in serum and tumor tissues of HCC patients and is associated with poor prognosis. Moreover, GS promotes HCC migration and invasion by EMT (Liu et al., 2020). Peri-tumoral hyperintensity in the hepatobiliary phase of gadoxetic acid-enhanced MRI (EOB-MRI) positively associated with high GS and organic anion transporter polypeptides (OATP)1B3 expression in the peri-tumoral zone. Peri-tumoral hyperintensity indicates a high potential for microscopic hepatic venous invasion (Yoneda et al., 2018).

Nevertheless, some studies also show that GS staining in HCC indicates a better prognosis. Wnt/β-catenin related makers (β-catenin, GS) positive HCC mark better differentiation, less portal vein invasion, and intrahepatic metastasis (Tsujikawa et al., 2016). β-catenin activation by fluorescence *in situ* hybridization and glutamine synthetase highly staining by immunohistochemistry demonstrates the character of well-differentiated HCC (Evason et al., 2013). GS-positive patients have reduced tumor-specific mortality and overall mortality (Dal Bello et al., 2010). The positive of glutamine synthetase indicates better survival for HCC patients treated with liver transplantation (Ataide et al., 2017). In mice transgenic the full length of hepatitis B virus X protein, EMT increases, but glutamine synthetase level decreases (Ahodantin et al., 2020).

Glutamine synthetase also correlates with the PD-1 expression and treatment response or is influenced by treatment. For instance, GS overexpression is significantly associated with low expression of PD-1 in HCC patients (Montasser et al., 2021). Lower GS staining predicts better OS and RFS for patients treated with adjuvant TACE after curative resection in HCC patients (Zhang et al., 2015). Glucocorticoid promotes GS expression by transcriptional and posttranscriptional levels in hepatoma cell lines (Gaunitz et al., 2002).

### 4.2 Other glutamine metabolism-related enzymes

Glutamate dehydrogenase (GLUD) serves as a catalyticase that drive L-glutamate towards α-KG and ammonia, and α-KG is a pivotal component of the tricarboxylic acid cycle (TCA cycle). hGLUD1 is highly expressed in HCC human samples and HepG2 cells; The proliferation of HepG2 cells is reduced by silencing hGLUD1, which is mediated by decreasing mitochondria-mediated apoptosis (Marsico et al., 2021). Moreover, Preoperative serum GLUD predicts high microvascular invasion (MVI) and poor overall survival for HCC patients after liver transplantation (Gong et al., 2021).

Glutamine metabolism-related genes are upregulated in the HCC cohort from the TCGA database. Among them, glutaminase (GLS) 1 is increasing in HCC and associated with the stemness of HCC cells, which is also associated with poor prognosis (Jin et al., 2020). Higher expression of GLS1 is positively correlated with poor differentiation, more lymphatic metastasis, advanced stage, more elevated serum AFP, and lower overall survival. GLS1 promotes HCC cell proliferation and could be inhibited by GLS1 inhibitors. The mechanism might relate to GLS1 inducing the activation of the AKT/GSK3β/CyclinD1 pathway (Xi et al., 2019). Conversely, both protein and mRNA levels of glutaminase (GLS) 2 display negatively associated with late stage, vascular invasion, tumor relapse, overall survival, and disease-free survival. Mechanically, GLS2 stabilizes Dicer by ubiquitination system; Induced Dicer promotes miR-34a maturation; mature miR-34a will repress snail expression, which is reported to facilitate HCC cells invasiveness and epithelial-mesenchymal transition (Kuo et al., 2016).

### 4.3 Metabolites

A “serum biomarker model” containing tryptophan, glutamine, and 2-hydroxybutyric acid based on capillary electrophoresis−time-of-flight mass spectrometry is established to diagnose HCC from non-HCC, which is confirmed to be an effective biomarker that compensatory for AFP (Zeng et al., 2014). ^1^H- nuclear magnetic resonance (NMR) metabolomics profiling is used to distinguish the early or late stage of HCC and find that glutamine decreases in the late stage of HCC with respect to the early stage of HCC (Casadei-Gardini et al., 2020). A study elucidates the metabolomics of HCC with different etiology by ^1^H-NMR and finds that HCC from NAFLD has high levels of glutamine/glutamate, which is also validated by increased expression of GS in immunochemistry and NMR-spectroscopy glutamine quantification. Nevertheless, HCC with cirrhosis acquires high levels of β-hydroxybutyrate, tyrosine, phenylalanine, and histidine (Teilhet et al., 2017). Serum-based metabolomics by ^1^H-NMR reveals that pyruvate, glutamine, and α-ketoglutarate are abundant in liver cirrhosis and HCC (Gao et al., 2009). Plasma phenylalanine and glutamine levels in patients with liver cirrhosis are associated with HCC occurrence in the next 3 years. Phenylalanine concentration positively correlates with HCC, and glutamine level is the opposite effectiveness (Liang et al., 2020). Another serum NMR-based metabolomics demonstrates that cirrhosis with large HCC has significant upregulation of glutamate, acetate, and N-acetyl glycoproteins. In contrast, the metabolic fingerprint for cirrhosis without HCC displays a high concentration of lipids and glutamine (Nahon et al., 2012). NAFLD-HCC with no or mild fibrosis predominantly overexpressed choline derivatives and glutamine by 1H-Nuclear Magnetic Resonance spectroscopy (Buchard et al., 2021).

### 4.4 Metabolite transporters

Solute Carrier Family 38 A1 (SLC38A1), a crucial glutamine transporter, is validated to be upregulated in HCC at both mRNA and protein by the Cancer Genome Atlas (TCGA) cohort and a Clinical Proteomic Tumor Analysis Consortium (CPTAC) cohort. Moreover, it is inversely correlated with CD8^+^ T cell infiltration (Liu et al., 2021). Solute carrier family 1 member 5 (SLC1A5), also terms as alanine–serine-cysteine transporter 2 (ASCT2), is a glutamine transporter. SLC1A5 is highly expressed in HCC and predicts poor prognosis, confirmed by multiple databases according to bioinformatics (Zhao et al., 2021). Glucose transporter GLUT1 and glutamine transporter ASCT2 are upregulated in HCC, and the high expression of GLUT1 and ASCT2 indicates poor OS and recurrence-free survival (RFS) (Sun et al., 2016).

## 5 Glutamine metabolism reprogramming in HCC

### 5.1 Glutamine as fuel for HCC proliferation

Increased glutamine uptake and more glutamine converts to the TCA cycle are confirmed in studies. Glutamate dehydrogenase (GLUD) serves as a catalyticase that drive L-glutamate towards α-KG and ammonia, and α-KG is a pivotal component of the TCA cycle. hGLUD1 is highly expressed in HCC human samples and HepG2 cells; The proliferation of HepG2 cells is reduced by silencing hGLUD1, which is mediated by decreasing mitochondria-mediated apoptosis (Marsico et al., 2021). Discoidin domain receptor 1 (DDR1) is highly expressed in HCC, which promotes glutamine metabolism as fuel by increasing GLUD1, GLS1, and SLC1A (glutamate transporter) in HCC (Lin et al., 2020). circGSK3B is confirmed to promote HCC cell proliferation and metastasis ability by increasing GLS (Li et al., 2020). SIRT4 localizes in mitochondria and regulates glutamine or lipid metabolism. SIRT4 is downregulation in mRNA and protein levels confirmed by human HCC samples; knockout or silence of SIRT4 will promote hepatocarcinogenesis *in vivo* and *in vitro*. Mechanically, SIRT4 inhibits the conversion from glutamine fuel to the TCA cycle. Decreasing glutamine catabolism results in a deficiency of ATP/ADP, leading to the activation of the LKB1/AMPKα/mTOR axis (Wang et al., 2019). High-mobility group box 1 gene (HMGB1) acts as competing endogenous RNAs (ceRNAs) for the mTORC2 component RICTOR, subsequently promoting the expression of RICTOR mRNA. The high expression of RICTOR will induce mTORC2-AKT-C-MYC activation that upregulates GS expression; on the other hand, GLUD will be enhanced as the release of inhibition signal from SIRT4 (Wei et al., 2021).

The HGF-MET axis is confirmed to stimulate glycolysis and glutaminolysis to function as a biogenetic source for HCC cell lines via inhibiting pyruvate dehydrogenase complex (PDHC) activating GLS. However, dephosphorylated MET-mediated autophagy compensates for sustaining biogenesis, leading to the treatment resistance of HGF-MET axis inhibitors or antibodies. Other autophagy blockers to HGF-MET axis inhibitors improve the therapeutic efficiency of HCC *in vitro* and *in vivo* (Huang et al., 2019). High expression of TGF-β in the HCC cell line demonstrates a mesenchymal-like morphology. Glutamine anaplerosis for the fuel compensation to the biosynthetic utility of TCA metabolites is confirmed in TGF-β highly expressed cell line. The mechanism related to TGF-β in the HCC cell line might be elevated glutamine transporter solute Carrier Family 7 Member 5 (SLC7A5) and GLS1 (Soukupova et al., 2017). However, in a doxycycline-regulated Myc transgenic model of HCC, glutamine transporter SLC1A5 is highly expressed, and GLS1/GLS2 is downregulated in both transcripts and protein, which indicates increased extracellular glutamine uptake to anabolic pathway other than fuel source for the TCA cycle (Dolezal et al., 2017). Chemo-resistance HCC cell lines display cancer stem cell-like phenotype with rising CSC markers, poorly developed mitochondrial network, and increasing telomerase activity. The chemo-resistance character is mediated by drug efflux caused by high expression of P-gp protein, which is an ATP-consuming process. However, glucose-dependent OXPHOS and glycolysis are decreasing, indicating a metabolic quiescent in chemo-resistance cell lines. An alternative source from the glutamine-OXPHOS pathway fuels the ATP. Co-treatment of mitochondria-specific antagonist metformin and glutamine-starving condition attenuates the drug efflux in chemo-resistance HCC cell lines (Lee et al., 2021). The details are shown in Figure 1.

### 5.2 The source of nitrogen

Glutamine metabolism supplies carbon and nitrogen sources for amino acid or nucleotide anabolism, as shown in Figure 1. Yap overexpression induces hepatomegaly and promotes carcinogen dimethylbenzanthracene (DMBA)-induced liver tumor formation by activating GLUL as a transcriptional factor. Elevated GLUL enhances glutamine accumulation, which provides sufficient nitrogen into nucleotide biosynthesis that accelerates liver and liver tumor proliferation (Cox et al., 2016). However, a study finds that the serine biosynthesis pathway (SSP) is activated, and cMyc expression is elevated during glucose or glutamine deprivation. Potential mechanisms might be related to cMyc-regulated enzymes like glutathione (GSH) and phosphoserine phosphatase (PSPH), which promote redox hemostasis for cancer cells and activate the serine biosynthesis pathway (Sun et al., 2015).

### 5.3 More glutamine converts to glutathione as an antioxidant in HCC

Glutathione-glutamine-glutamate metabolism aberration is involved in the process of hepatic tumorigenesis (Chen et al., 2019). Glutamine uptake in HCC is not predominantly as carbon or fuel for the TCA cycle but for increasing the conversion of glutamine into glutamate, thereby converting more glutamate into glutathione. Glutathione functions as an antioxidant that prevents oxidative damage to cancer cells. In an HCC mice model induced by co-transfection of c-Myc/h-Ras, glutamine synthetase (GS), expressed in pericentral hepatocytes in a healthy liver, is absent within the tumor in the c-Myc/h-Ras mice model. Glutamate-cysteine ligase catalytic subunit (Gclc) increases, and GLUD1 decreases in the c-Myc/h-Ras mice model, which indicates that more glutamate converts to glutathione other than α-ketoglutarate (Serra et al., 2022). Metabolic competition for glutamine is validated to impair hepatocellular tumorigenesis. Mitochondrial Pyruvate Carrier (MPC) is located in the inner membrane of mitochondria and serves as a pyruvate transporter from the cytoplasm into mitochondria. This crucial metabolic crossroad links glycolysis and the tricarboxylic acid (TCA) cycle. MPC is elevated in human HCC samples validated by The Cancer Genome Database (TCGA). Liver-specific MPC depletion in N-nitrosodiethylamine (DEN) plus carbon tetrachloride (CCl4) induced HCC mouse model attenuates HCC tumorigenesis. The underlying mechanism is correlated with the glutamine competition; briefly, disrupting MPC causes decreasing pyruvate flux from the cytosol and, subsequently, glutamine metabolic into α-ketoglutarate to compensate for reduced pyruvate uptake caused by MPC depletion in the TCA cycle. Conversely, glutathione synthesis confirmed to protect cancer cells from reactive oxygen species (ROS) damage, will be competitive as glutamine consumption for the TCA cycle (Tompkins et al., 2019), as shown in Figure 1.

GLS1 is highly expressed in HCC patients and cell lines. Upregulated GLS1 promotes the production of glutamate, the precursor of GSH, which serves as the main cellular antioxidant. The reduction of ROS will enhance the translocation of β-catenin, upregulating the stemness-related genes (KFL4, NANOG, OCT4, SOX2, CD13, and CD44) in HCC (Li et al., 2019). Oxoglutarate dehydrogenase-like (OGDHL) is one of the rate-limiting enzymes of oxoglutarate dehydrogenase complex (OGDHC) in the canonical TCA cycle. OGDHL is verified to be low expressed in the TCGA database, Gene Expression Omnibus (GEO) database, and FUDAN database. The downregulation of OGHDL is associated with HCC progression, poor prognosis, and recurrence. Mechanically, low OGDHL reduces the forward TCA cycle for glucose oxidation. Conversely, reductive carboxylation of α-ketoglutarate (αKG) is facilitated to promote lipogenesis. Moreover, increasing glutamine consumption enhances antioxidative function to protect against oxidative stress in HCC, inhibiting glutamine metabolism could improve sorafenib resistance (Dai et al., 2020). Glutamine deprivation promotes a shift of glycolysis towards oxidative phosphorylation (OXPHOS) in HCC cell lines. The mechanism underlying this phenomenon is glutamine deprivation inducing increased NADP1/NADPH ratio and GSH/GSSG ratio that causes an elevation of cellular reactive oxygen species (ROS); increased ROS enhances the overexpression of retinoic acid-related orphan receptor alpha (RORα), and RORα mediates reprogramming of glucose metabolism towards OXPHOS rather than glycolysis by attenuating pyruvate dehydrogenase kinase 2 (PDK2) and lactate dehydrogenase A (LDHA) (Byun et al., 2015). However, a study finds that the serine biosynthesis pathway (SSP) is activated, and cMyc expression is elevated during glucose or glutamine deprivation. The potential mechanism might be related to cMyc-regulated enzymes like glutathione (GSH) and phosphoserine phosphatase (PSPH), which promote redox hemostasis for cancer cells and activate the serine biosynthesis pathway (Sun et al., 2015). Selected sorafenib-resistant HCC cell lines display higher reductive glutamine metabolism than parental cell lines. Mechanisms, increased expression of PPARδ in sorafenib-resistant HCC induces higher expression of enzymes that catalyze glutamine metabolism and pentose phosphate pathway, incredibly reductive glutamine metabolism in the TCA cycle. The redox homeostasis that protects from oxidative stress will be enhanced by more glutamate synthesis from glutamine, and more reductive glutamine metabolism promotes lipid biosynthesis that promotes HCC proliferation. Moreover, more pentose phosphate pathway products facilitate HCC proliferation (Kim et al., 2017).

### 5.4 Glutamine-related metabolites activate the mTORC signaling pathway

GS and mTORC are highly expressed in the β-Catenin gene mutated mouse model with HCC or HCA. In clinical samples, cases with CTNNB1 mutation show intense GS staining, and GS strongly positive cases display high staining for p-mTOR-S2448. In normal mice, GS and p-mTOR-S2448 co-staining in pericentral hepatocytes. The mechanism of β-Catenin mutation-related HCC is induced by β-Catenin -GS-mTORC1 axis. Briefly, CTNNB1-mutation induces GS transcription by β-Catenin translocation and activating transcription factors. The elevated GS will catalyze more glutamate to glutamine; subsequently, glutamine promotes p-mTOR activation, promoting the proliferation of HCC (Adebayo Michael et al., 2019), as shown in Figure 1. Liver receptor homolog 1 (LRH-1) increases in the DEN-induced HCC mouse model. LRH-1 knockout mice display fewer tumors than wild ones. Mechanisms, LRH-1 enhances noncanonical glutamine metabolism by increasing GLS2, which catalyzes Gln to Glu, then Glu converts to α-KG by glutamate pyruvate transaminase 2 (Gpt2). Subsequently, α-KG will modulate mTORC1, facilitating cell proliferation (Xu et al., 2016). c-Myc-dependent hepatocarcinogenesis requires mTORC1 pathway activation to acquire the property of tumorigenesis. Mechanisms, amplification, or activation of c-Myc as a transcriptional factor leads to high expression of amino acid transporters SLC1A5/SLC7A6; increased amino acid transporters are responsible for more amino acid uptake, especially glutamine. After that, increasing amino acids results in mTORC1 activation, a typical pathway that induces cell proliferation (Liu et al., 2017). Nine unique short hairpin RNA (shRNA) vectors and six unique CRISPR-Cas9 vectors are used to repress the expression of glutamine transporter (ASCT2), and L-Type Amino Acid Transporter 1 (LAT1), glutamine or leucine transportation is restrained. However, the mTORC1 pathway and cell proliferation are unchanged (Bothwell et al., 2018).

### 5.5 Glutamine-related metabolites regulate other metabolisms or signaling pathways

**FIGURE 2:**
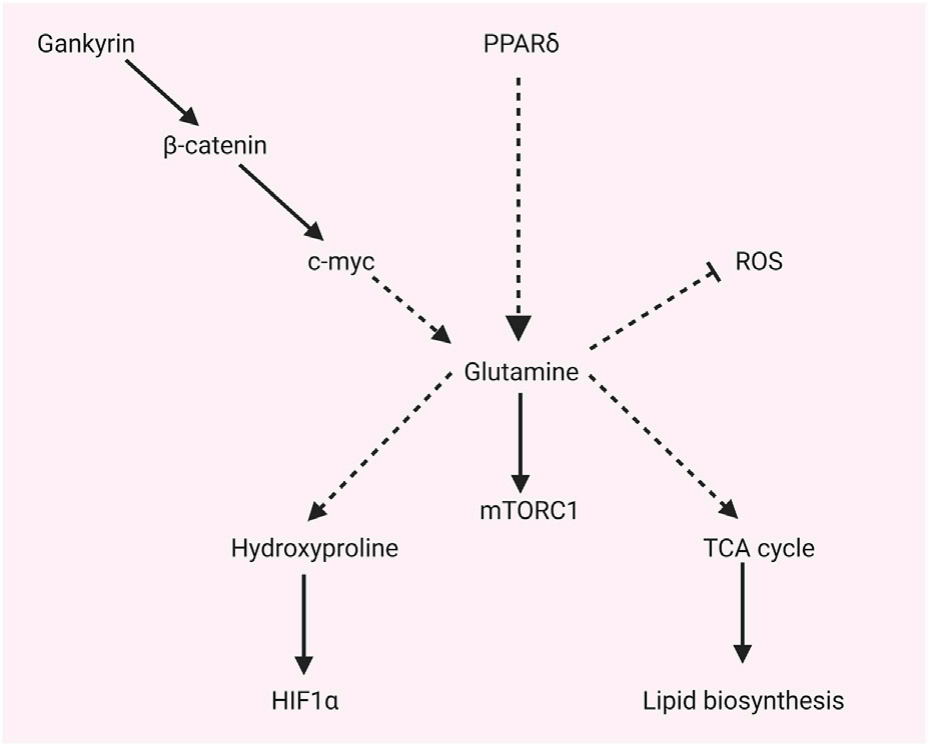
Glutamine-related metabolites regulate other metabolisms or signaling pathways.

As shown in Figure 2, glutamine and related metabolites not only activate the mTORC signaling pathway, but also regulate other metabolisms or signaling pathways. The downstream amino acid of glutamine metabolism, hydroxyproline, is confirmed to play a crucial role in promoting a hypoxic response in HCC. Hydroxyproline is accumulated in HCC according to global metabolic profiling. A high level of hydroxyproline is correlated with elevated AFP and poor prognosis. Mechanically, hydroxyproline blocks hydroxylation of HIF1α and attenuates the binding of HIF1α to tumor suppressor proteins during hypoxia to increase the HIF1α expression. Exogenous hydroxyproline could recover the effect of Myc or ALDH18A1 knockdown, which inhibits the glutamine–hydroxyproline metabolism or proline metabolic pathway. Moreover, hydroxyproline inhibition could attenuate the sorafenib resistance under hypoxia (Tang et al., 2018). Activated mTORC1 induced by knockout of tumor suppressor gene tuberous sclerosis complex (TSC) promotes glutaminolysis, leading to glutamine depletion. Fibroblast growth factor 21 (FGF21) will be activated by glutamine depletion stress by activating peroxisome proliferator–activated receptor γ coactivator-1α (PGC-1α). Elevated FGF21 results in reduced liver triglyceride content, decreased locomotor activity, and body temperature (Cornu et al., 2014). Selected sorafenib-resistant HCC cell lines display higher reductive glutamine metabolism than parental cell lines. Mechanisms, increased expression of PPARδ in sorafenib-resistant HCC induces higher expression of enzymes that catalyze glutamine metabolism and pentose phosphate pathway, incredibly reductive glutamine metabolism in the TCA cycle. The redox homeostasis that protects from oxidative stress will be enhanced by more glutamate synthesis from glutamine, and more reductive glutamine metabolism promotes lipid biosynthesis that promotes HCC proliferation. Moreover, more pentose phosphate pathway products facilitate HCC proliferation (Kim et al., 2017). Uncoupling protein (UCP) 2 is a type of the mitochondrial carrier family involved in metabolic disorders. UCP2 promotes glutaminolysis to decrease glutamine-derived C4 metabolite accumulation in mitochondria. However, it reduces the oxidation of glucose (Vozza et al., 2014). Liver-specific miR-122 is validated to play a critical role in glutamine metabolism. miR-122 is negatively correlated with the expression of GLS, according to the TCGA database. The liver-specific knockout of miR-122 promotes glutaminolysis but inhibits gluconeogenesis in mice by decreasing targets of GLS and SLC1A5 (Sengupta et al., 2020). Gankyrin is a seven ankyrin-repeat domains protein. It promotes HCC tumorigenesis, metastasis, and sorafenib or regrafenib resistance. Mechanisms, it stabilizes RNA-binding protein HuR, which subsequently stabilizes β-catenin mRNA and increases its expression. β-catenin could promote c-myc expression, which regulates glycolysis and glutaminolysis by transcriptionally activating crucial enzymes such as GLUT1, ASCT2, HK2, PKM2, LDHA, and GLS1. Gankyrin displays the characteristics that facilitate glycolysis and glutaminolysis, which c-myc inhibitors could abolish *in vitro* and *in vivo*. High Gankyrin and high β-catenin indicate poor prognosis with low overall survival (Liu et al., 2019).

## 6 Glutamine metabolism targeting therapy

**FIGURE 3:**
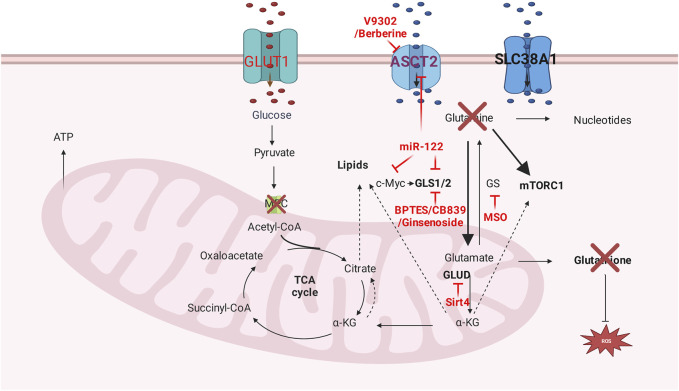
Glutamine metabolism targeting therapy. Glutamine transporters inhibitors, glutamine deprivation, and glutamine metabolism related enzyme inhibitors are dominant methods that targeting glutamine metabolism.

Glutamine metabolism targeting therapy includes glutamine deprivation, related enzyme inhibitors, and transporters inhibitors, as shown in Figure 3. Glutamine deprivation impairs severe metabolism reprogramming in a poorly differentiated cell line, which results in kinase inhibitors resistance as increased phosphorylation of extracellular signal-regulated kinase (Nwosu et al., 2020). Additional glutamine supplement displays dose-dependent anti-tumor effects in HepG2 and Huh7 cell lines and increases the sensitivity of histone deacetylase inhibitor vorinostat in both cell lines (Hassan et al., 2021). 30% of glutamine is metabolized to produce glutamate in the cytoplasm, which functions as the substrate of nucleotide synthesis. Inhibition of glutamate excretion will perturb cell growth *in vitro* (Nilsson et al., 2020).

GLS loss or GLS-specific inhibitor bis-2-(5-phenylacetamido-1,3,4-thiadiazol-2-yl)ethyl sulfide (BPTES) attenuates tumor progression and prolonged survival in Myc-driven HCC mouse model (Xiang et al., 2015). Noncompetitive allosteric GLS1 inhibitor CB-839 monotherapy displays insufficient anti-cancer effectiveness in HCC cell lines. Nevertheless, ASCT-2 inhibitor V-9302 could be synergistic with CB-839 to function as anti-tumor efficacy. Mechanically, the combination of V-9302 and CB-839 disrupts ROS balance by decreasing important antioxidant-glutathione (GSH). Moreover, reducing glutamine intake in TCA results in the reduction of NADPH, which serves as GSH biosynthesis (Jin et al., 2020). Ginsenoside Rk1 demonstrates anti-tumor effectiveness by downregulating GLS1, decreasing GSH, and subsequently accumulating ROS (Lu et al., 2022); and there are two clinical trials that use Ginsenoside in hepatocellular carcinoma(NCT01717066, NCT04523467). Dihydroartemisinin (DHA) induces oxidative stress in cancer cells by increasing intracellular reactive oxygen species (ROS). Glutaminase (GLS) 1 increases the production of antioxidants like GSH by generating the precursor glutamate. The combination of GLS1 inhibitor and DHA has synergistic antitumor efficacy in HCC by increasing ROS and decreasing GSH (Wang et al., 2016).

Berberine inhibits the proliferation of HCC cell lines by suppressing c-myc-induced glutamine transporter SLC1A5, subsequently decreasing glutamine uptake (Zhang et al., 2019). Glutamine depletion by bacterial enzyme Crisantaspase and/or GS inhibitor methionine-L-sulfoximine (MSO) hinders the tumor growth of human HCC xenografts induced by CTNNB1-mutated HCC cell lines (Chiu et al., 2014). β-catenin-mutated HCC cell line is more sensitive to glutaminolysis drug-asparaginase (ASNase) (Tardito et al., 2011). Tigecycline, an electron transport system (ETS) inhibiting antibiotic, is effective in both sorafenib-resistant advanced-stage HCC *in vitro* and in xenograft *in vivo*. The mechanism disrupts the mitochondrial ETS complex biogenesis and impairs glutamine oxidation (Meßner et al., 2020). Oral nutritional supplement (ONS) that contains β-hydroxy-β-methyl butyrate (HMB), L-arginine, and L-glutamine serves as effective prophylactic supplementation for Hand-foot skin reaction (HFSR) caused by sorafenib in advanced HCC patients (Naganuma et al., 2019).

## 7 Conclusion

Metabolism reprogramming plays a pivotal role in HCC; It’s not only the outcome of HCC initiation or progression but also the mainstay of factors causing HCC occurrence or promoting HCC metastasis. The components of glutamine metabolism are altered in HCC, indicating biomarkers’ potential roles, including related metabolism-related enzymes, metabolites, and metabolites’ transporters. The glutamine metabolism reprogramming support HCC cancer cells as carbon and nitrogen sources; It provides antioxidant for HCC survival; It activates the mTORC signaling pathway to support tumor cell proliferation. Targeting glutamine reprogramming, including glutamine deprivation, related enzyme inhibitors, and transporters inhibitors, therefore simultaneously limit energy availability and increase oxidative stress, demonstrate potential therapy in HCC; However, cancers can evade this metabolic trap by reprograming their metabolism (Halama and Suhre, 2022), which is confirmed in ClinicalTrial. Therefore, the effectiveness that rely solely on of glutamine inhibition is limited; cotreatment with other strategies might constitute an attractive and promising option for HCC patients.
